# Comparison of two bovine serum pregnancy tests in detection of artificial insemination pregnancies and pregnancy loss in beef cattle

**DOI:** 10.1371/journal.pone.0211179

**Published:** 2019-01-23

**Authors:** Emmalee J. Northrop, Jerica J. J. Rich, Jim R. Rhoades, George A. Perry

**Affiliations:** 1 Department of Animal Science, South Dakota State University, Brookings, SD, United States of America; 2 IDEXX Laboratories, Westbrook, ME, United States of America; University of Illinois, UNITED STATES

## Abstract

Blood tests for early detection of pregnancy in cattle based on pregnancy-associated glycoproteins (PAGs) are commercially available. The objective of these studies were to compare the accuracy of blood tests to transrectal ultrasonography in detecting AI pregnancies, and to compare the accuracy of blood tests in predicting pregnancy loss. Beef cattle from 6 herds were synchronized using a recommended CIDR based protocol (Study 1: n = 460; Study 2: n = 472). Pregnancy status was determined by transrectal ultrasonography between days 28–40 following AI, blood samples were collected at this time. In study 2 a final pregnancy determination was performed at the end of the breeding season to determine pregnancy loss. Each serum sample was examined for PAG concentrations using a microtiter plate reader and/or scored by two technicians blind to pregnancy status and pregnancy loss. For study 1 Cohen’s kappa statistics were calculated to assess the agreement between each test and transrectal ultrasonography. For study 2 data was analyzed using the GLIMMIX procedure of SAS with herd as a random effect, and loss, age, and their interaction included in the model. Agreement was good to very good for each test. There was no difference (*P* = 0.79) in sensitivity, but a difference (*P*<0.01) in specificity of the assays (88%, 64%, 87%, 90%) and in the overall percent correct (93%, 84%, 93%, 93%). There was an effect of pregnancy loss (*P* = 0.04), age (*P* = 0.0002), and their interaction (*P* = 0.06) on PAG concentrations. In conclusion both pregnancy tests were accurate at detecting AI pregnancies, and were in very good agreement with transrectal ultrasonography. Both tests detected differences in PAGs among females that maintained and lost pregnancy; however, prediction proved to be difficult as most females were above the threshold and would have been considered pregnant on the day of testing.

## Introduction

Early pregnancy diagnosis is critical for maximizing herd productivity in the cattle industry. Current methods for pregnancy diagnosis include: observation for return to estrus, rectal palpation, transrectal ultrasonography, and blood tests for specific antigens. These blood tests include detection of pregnancy-associated glycoproteins (PAGs) to determine pregnancy status as early as 28 days post-breeding. The PAG family is comprised of 20 individual proteins, and two dozen genes. Pregnancy-associated glycoproteins are aspartic proteinases that originate from binucleate cells of the embryonic trophoblast [1]. Binucleate cells fuse with uterine epithelial cells and release their secretory products into maternal circulation [2]. Previous research determined that there are large variations in PAG production among individual females [3]. However, previous research examined serum concentrations of Pregnancy Specific Protein B [PSPB] throughout gestation using a radioimmunoassay [4]. They determined that serum concentrations were approximately 3 ng/ml from day 42 to day 70. There was a linear increase from day 70 to day 150, when concentration reached approximately 19 ng/ml in serum. Following day 150, there was a rapid increase until day 262 of gestation, when concentration reached 73 ng/ml. By parturition, the concentration increased to approximately 495 ng/ml. Previous research has determined that PAGs have a long half-life due to carbohydrate and sialic acid content and can be detected in the blood 80–100 days postpartum [3]. Specifically, the half-life of PSPB in postpartum cows after calving was determined to be 7.3 to 8.4 days [5,6].

Artificial insemination is a reproductive technology that allows cattle producers to improve genetics, increase weaning weights, calf uniformity, herd productivity, and reproductive performance, while reducing bull related costs. The ablity to identify AI sired calves from bull bred calves is critical for cow-calf operations. The common practice for identifying these calves is to perform an initial pregnancy diagnosis by transrectal ultrasonography 30 days post breeding to determine AI calves, then perform a final pregnancy diagnosis at the end of the breeding season to determine bull bred calves and any pregnancy loss that may have occurred. However, this method requires specialized equipment and trained personnel to determine the difference between an AI pregnancy and a bull bred pregnancy [7].

Among cattle, fertilization rates are reported to be around 90%, while calving rates fall within the range of 50 to 60% [5], and much of this pregnancy loss occurs within the first three weeks of pregnancy [8–12]. Previous research has further elucidated that embryonic loss is most prevalent between days 16 and 29 [13,14]. However, this time period encompasses conceptus elongation, maternal recognition of pregnancy, and the beginning of attachment to the uterus. Further research is necessary to determine specifically when embryonic loss is occurring, and the mechanism by which it happens in cattle. The hypotheses of these studies were that 1) PAG blood tests would be similar in accuracy when compared to transrectal ultrasonography in detecting AI pregnancies around day 28 of gestation, and 2) the PAG tests would be able to detect differences in PAG concentrations between beef cows and heifers that experienced pregnancy loss and ones that did not. Therefore, the objective of these studies were 1) to compare the accuracy of blood tests for PAGs to transrectal ultrasonography in their ability to determine pregnancy around day 28 (detection of AI sired fetuses), and 2) to compare the accuracy of blood tests in predicting pregnancy loss in beef cattle.

## Materials and methods

All procedures were approved by the South Dakota State University Institutional Animal Care and Use Committee.

### Experimental design

In study 1, beef cows and heifers (n = 460; 238 cows and 222 heifers) from 6 cooperator herds in South Dakota were synchronized using a recommended CIDR based protocol. All cows and heifers were maintained separate from bulls for 10 to 15 days after AI. Pregnancy status was determined via transrectal ultrasonography between 28 and 40 days following AI using an Aloka 500V ultrasound with a 7.5 MHz transrectal linear probe (Aloka, Wallingford, CT).

In study 2, beef cows and heifers (n = 984) from 6 different cooperator herds in South Dakota were synchronized using a recommended CIDR based protocol. Pregnancy status was determined via transrectal ultrasonography between 28 and 42 days following AI using an Aloka 500V ultrasound with a 7.5 MHz transrectal linear probe (Aloka, Wallingford, CT). A final pregnancy determination was performed at > 30 days after the end of the breeding season to determine pregnancy loss.

### Blood sampling

In study 1, blood samples (10 mL) were collected from all beef heifers and cows at time of pregnancy determination (n = 460 between day 28 and 40 of gestation) by jugular venipuncture into 10 mL vacutainer tubes (Fisher Scientific, Pittsburgh, PA). In study 2, blood samples (10 ml; n = 472; 247 heifers, 225 cows [2–11 years old]) were collected from only pregnant animals. Blood was allowed to coagulate at room temperature, stored at 4˚C for 24 hours, and centrifuged at 1,200 x *g* for 30 minutes at 4˚C. Serum was collected and stored at -20˚C until pregnancy tests were performed.

### Bovine pregnancy tests

Each serum sample was examined in duplicate using the IDEXX Laboratories (Westbrook, ME) Bovine Pregnancy Test (BPT) and the IDEXX Laboratories (Westbrook, ME) Rapid Visual Pregnancy Test (RVPT) according to the manufacturer’s instructions. The results from both the Bovine Pregnancy Test (BPT) and the Rapid Visual Pregnancy Test (RVPTOD) were analyzed using a Molecular Devices SpectraMax 190 microtiter plate reader (San Jose, California). The BPT was analyzed at 450nm and at 650nm, while the RVPT was analyzed at 650nm. Additionally, the RVPT was individually scored and evaluated by two technicians blind to pregnancy status and pregnancy loss. The scoring system consisted of yes/no (RVPTY/N) and a numerical value (0–3) based on color compared to the negative and positive controls (RVPTscore), where a score of 0 had the same or less color than the negative control, a score of 1 had slightly more color than the negative control, a score of 2 had slightly less color than the positive control, and a score of 3 had the same or more color than the positive control.

### Statistical analysis

For study 1, data were analyzed to determine specificity, sensitivity, positive predictive value, negative predictive value, and percent correct between each blood test and transrectal ultrasonography using the GLIMMIX procedure of SAS with herd as a random effect. Cohen’s kappa statistics were calculated to assess the agreement of the tests and transrectal ultrasonography using the FREQ procedure in SAS, the Kappa scoring scale is as follows: 0.80–1.00 = Very good, 0.60–0.80 = Good, 0.40–0.60 = Moderate, 0.20–0.40 = Fair, and <0.20 = Poor. The logistic procedure in SAS was used to create Receiver Operating Characteristic curves (ROC) for both the BPT and the RVPTOD tests, the scoring scale for the ROC curves is as follows: 0.90–1.00 = Excellent, 0.80–0.90 = Good, 0.70–0.80 = Fair, 0.60–0.70 = Poor, and 0.50–0.60 = Fail.

In study 2 (n = 472; 247 heifers, 225 cows), data was analyzed using the GLIMMIX procedure of SAS with pregnancy loss, age (heifer/cow), and their interaction included in the model. Herd was included as a random variable. Differences were considered to be significant when *P* ≤ 0.05 and a tendency when *P* > 0.05 but *P* ≤ 0.10.

## Results

### Study 1

**Fig 1:**
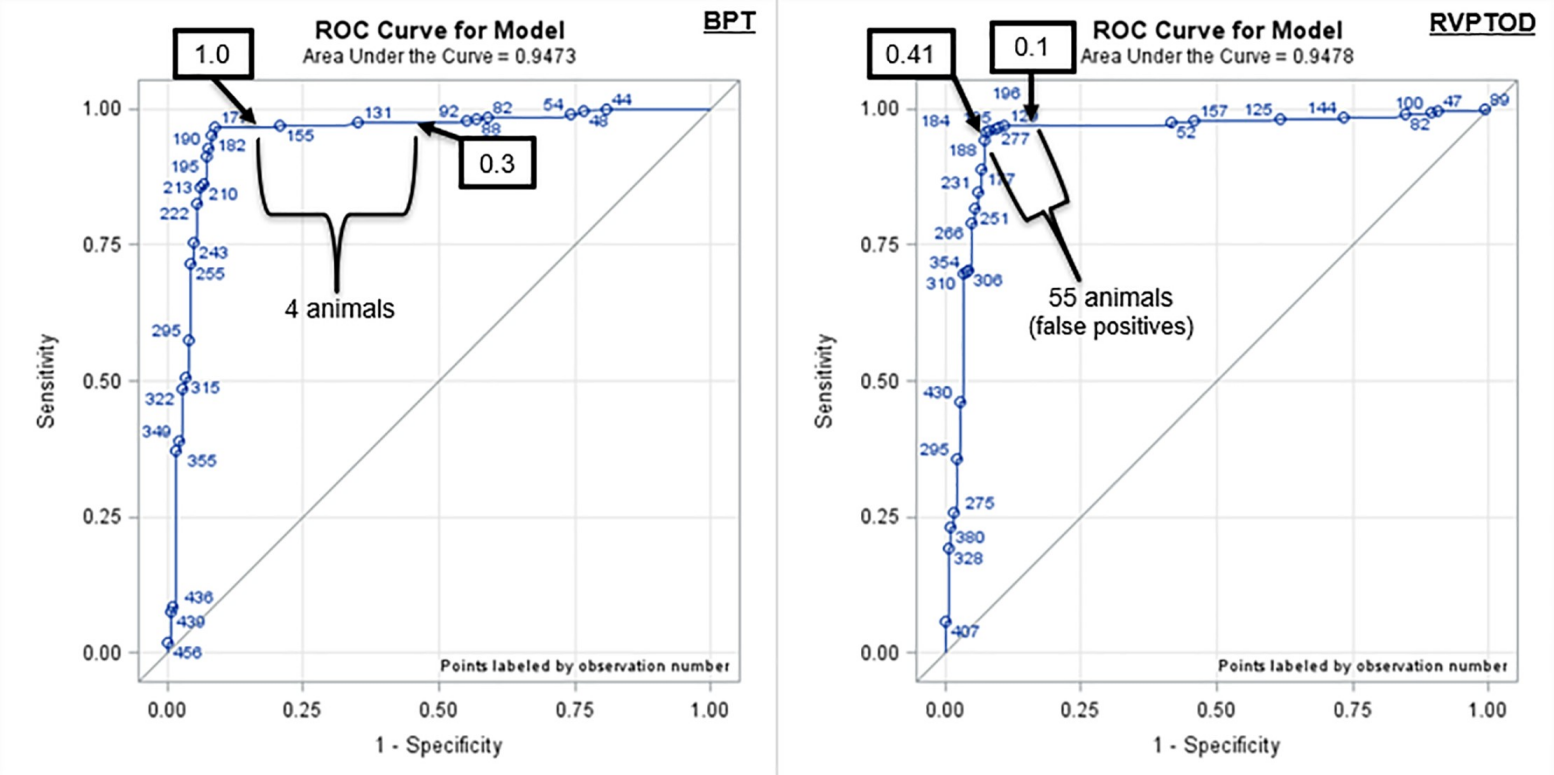
Receiver operator characteristic (ROC) curves for the Bovine Pregnancy test (BPT) and the Rapid Visual Pregnancy Test read on the microtiter plate reader (RVPTOD). The ROC curve is a graphical depiction of the true positive rate and the false positive rate when an increasing cut-off for the test is applied. If both the true positive rate and the false positive rate decrease when the cut-off is increased then the graph has a diagonal line and the test is not useful. Deflection of the line to the left of the center is an indication of a useful test because it has a relatively high true positive rate and low false positive rate at a given cut-off. With the BPT a cut-off of 0.3 is considered pregnant and a cut-off of 1 is considered definitely pregnant, in study 1 only 4 beef cows and heifers fell between these values. With the RVPTOD test, when a cut-off value of anything darker than the negative control (0.1) was used 55 beef cows and heifers that were determined to be not pregnant by transrectal ultrasonography were called pregnant, thus using a cut-off of 0.41 more accurately reflected the cut-off between pregnant and nonpregnant animals.

There was no difference (*P* ≥ 0.67) in the sensitivity (pregnant correctly diagnosed pregnant) of the tests (97%, 97%, 97%, and 96% for BPT, RVPTOD, RVPTscore, and RVPTY/N) and in the negative predictive value (95%, 95%, 95%, and 93% for BPT, RVPTOD, RVPTscore, and RVPTY/N) between the respective test and transrectal ultrasonography. There was a difference (*P* < 0.01) in the specificity (non-pregnant correctly diagnosed non-pregnant) of the tests (88%, 64%, 87%, and 90% for BPT, RVPTOD, RVPTscore, and RVPTY/N), in the overall percent correctly identified (93%, 84%, 93%, and 93% for BPT, RVPTOD, RVPTscore, and RVPTY/N), and in the positive predictive value (93%, 80%, 92%, and 94% for BPT, RVPTOD, RVPTscore, and RVPTY/N) between the respective test and transrectal ultrasonography. Agreement based on kappa scores was very good for the BPT (0.86), RVPTscore (0.85), and RVPTY/N (0.86) in comparison to transrectal ultrasonography **(Table 1)**. However, there was only good agreement for RVPTOD (0.64) when the OD threshold was set at any reading darker than the negative control. Receiver operating characteristic curves were used to evaluate the diagnostic ability of the two pregnancy tests. The area under the curves was 94.7% and 94.8% for the BPT and RVPTOD, respectively (**Fig 1**).

**Table 1:** Study 1: Agreement between blood tests for determining pregnancies on day 28 to 40 after AI compared to with transrectal ultrasonography on day 28 to 40 after AI.

	BPT	RVPTOD	RVPTscore	RVPTY/N	P-Value
Sensitivity^1^	97%	97%	97%	96%	0.7906
Specificity^2^	88%^a^	64%^b^	87%^a^	90%^a^	<0.0001
Positive Predictive Value^3^	93%^a^	80%^b^	92%^a^	94%^a^	<0.0001
Negative Predictive Value^4^	95%	95%	95%	93%	0.6689
Percent Correct^5^	93%^a^	84%^b^	93%^a^	93%^a^	0.0003
Kappa Score^6^	0.86	0.67	0.89	0.86	

^1^Sensitivity: Ability to correctly identify pregnant beef cows and heifers

^2^ Specificity: Ability to correctly identify non-pregnant animals

^3^ Positive Predictive Value: Likelihood a pregnant animal was called pregnant

^4^ Negative Predictive Value: Likelihood a non-pregnant animal was called non-pregnant

^5^ Percent Correct: Percent of beef cows and heifers that were correctly identified as pregnant or non-pregnant

^6^ Kappa Score: Good agreement was achieved at 0.60–0.80, and very good agreement was achieved at 0.80–1.00.

^ab^Values within a row having different superscripts are different at the P-Value specified.

BPT = Blood Pregnancy Test; RVPTOD = Rapid Visual Pregnancy Test read on a microtiter plate reader; RVPTscore = Rapid Visual Pregnancy Test visually scored 0 to 3 based on controls; RVPTY/N = Rapid Visual Pregnancy Test visually called yes pregnant or no open based on controls

### Study 2

**Fig 2:**
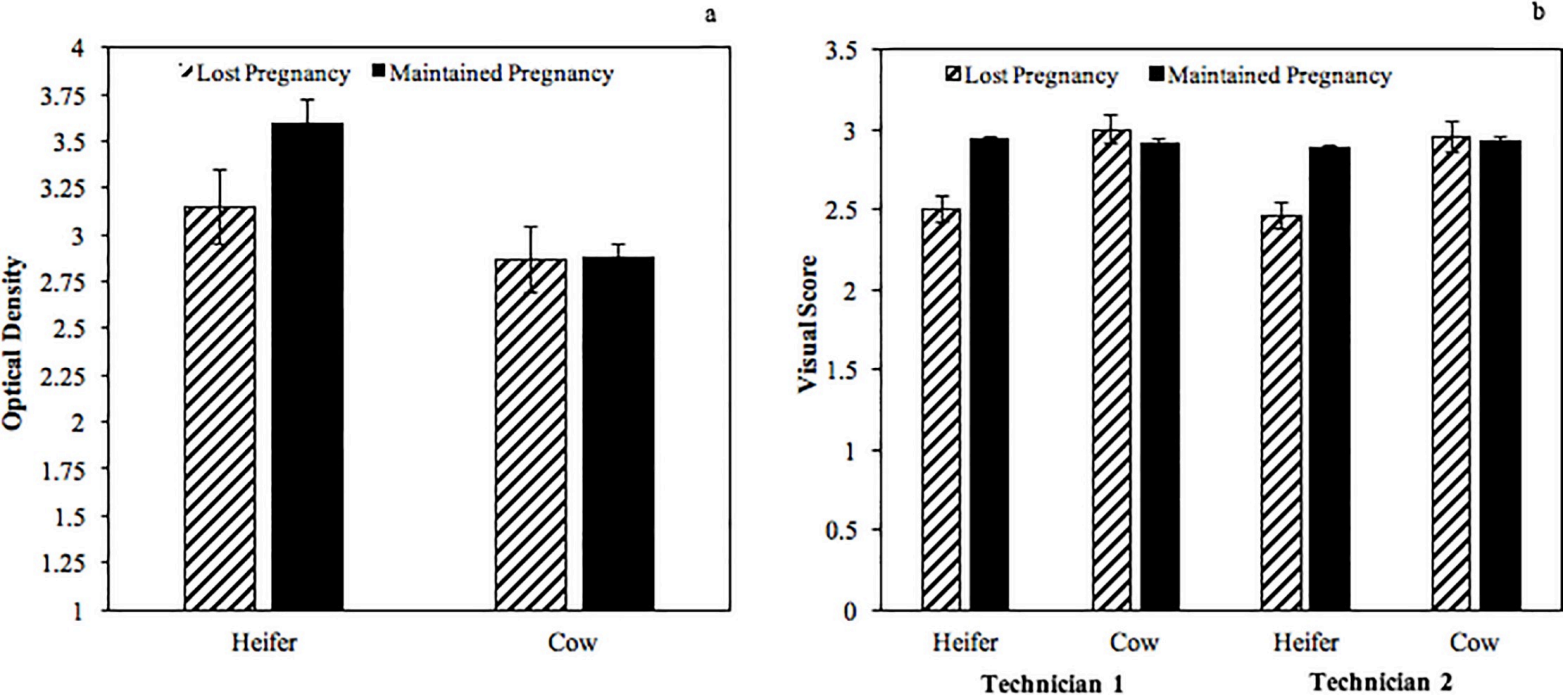
Comparison of optical density readings for the bovine pregnancy test (a) and rapid visual test scores (b) for beef cows and heifers that did and did not lose their pregnancy. Retrospectively, optical densities for beef females that lost pregnancies were lower than beef females that maintained pregnancies (*P* = 0.04), beef heifers had increased optical densities compared to beef cows (*P* < 0.001), and there was a tendency for an age by loss interaction (*P* = 0.06). In addition, both technicians independently scored beef females that lost pregnancies lower than beef females that maintained pregnancies (*P* < 0.001), heifers scored lower than cows (*P* <0.0001), and there was an age by loss interaction (*P* < 0.001).

There was a total of 28 (5.7%) beef cows and heifers that lost a pregnancy at some point between the first and second pregnancy diagnosis (after day 28). The BPT indicated decreased circulating concentrations of PAGs among beef females that lost their pregnancy (*P* = 0.04; 3.00 ± 0.14 vs 3.24 ± 0.09 OD). The BPT also indicated increased circulating concentrations of PAGs among heifers compared to cows (*P* = 0.0002; 3.37 ± 0.15 vs 2.87 ± 0.10 OD). There was a tendency for an age by loss interaction (*P* = 0.06; Fig 2A). For the RVPTscore, both technicians visually scored beef females that lost their pregnancy lower than beef females that maintained their pregnancy (*P* < 0.006; 2.75 ± 0.06 vs 2.93 ± 0.01 and 2.71 ± 0.07 vs 2.91 ± 0.02 for technician 1 and 2, respectively). Contrary to BPT results, both technicians visually scored heifers lower than cows using the RVPT (*P* < 0.0001; 2.72 ± 0.04 vs 2.96 ± 0.05 and 2.67 ± 0.05 vs 2.94 ± 0.05 for technician 1 and 2, respectively). There was an age by loss interaction (*P* < 0.0009; Fig 2B). Of the beef cows and heifers that lost their pregnancy only two were identified as not pregnant by the RVPT, and of the beef cows and heifers that maintained their pregnancy only one was falsely identified as not pregnant (0.2%).

## Discussion

The most common method for pregnancy detection in cattle is by a trained professional using rectal palpation [15] or transrectal ultrasonography [7], however, there is increased demand for more cost effective and easier methods such as chemical pregnancy detection. These blood tests detect Pregnancy Associated Glycoproteins (PAGs) which are produced by binucleate trophoblast cells. Binucleate trophoblast cells originate from mononucleate trophoblast cells by acytokinetic mitoses [16]. These binucleate cells represent about 15–20% of trophectodermal cells at the beginning of implantation and throughout pregnancy in ruminants [17]. These cells migrate and fuse with maternal endometrial epithelial cells and deliver granules via trinucleate cells and syncytial plaques into maternal circulation [18]. They cannot fuse with maternal endometrial epithelial cells until after attachment begins. The bovine conceptus begins attaching to the uterine wall around day 19, with adhesion occurring between days 21 and 22 [19]. By day 24, interdigitation has occurred [20], and by day 26 vascularization of the trophoblast has begun [17]. By day 28, areas of cuboidal uninucleated cells with a microvillar border have completed interdigitation [17].

The initial objective of this experiment was to determine the efficiency of a blood pregnancy test compared to transrectal ultrasonography to determine pregnancies that resulted from an AI service compared to a natural service when AI pregnancies were between 28 and 40 days and natural service pregnancies were less than 25 days. Based on ROC curves both the BPT and the RVPT were excellent tests. However, the RVPTOD was less sensitive, with decreased percent correct, and decreased positive predictive values compared to the BPT, RVPTscore, and RVPTY/N. In explaining these differences, it is important to acknowledge the manufacturer’s instructions, the BPT was designed to be conducted in a laboratory and analyzed on a microtiter plate reader, whereas the RVPT was designed to be conducted out of the laboratory and analyzed by the human eye. In this study, the RVPT was analyzed both by human eye and by a microtiter plate reader to determine if a microtiter plate reader would increase the accuracy of the test. In analyzing the RVPT this way the microtiter plate reader was set to determine any reading darker than the negative control as a positive/pregnant test. For the BPT only samples with an OD reading greater than 0.3 are considered pregnant and any samples with an OD reading greater than 1.000 were considered definitely pregnant. Using these values only four beef cows and heifers fell within that range. Conversely, for the RVPT, the greatest negative control value across all the plates was 0.1 OD, however, by analyzing the RVPT ROC curve the break occurs or should be considered at the reading of 0.41 OD, and 55 beef cows and heifers fall between 0.1 and 0.41 OD. All 55 beef cows and heifers were classified as non-pregnant by transrectal ultrasonography, but pregnant by the RVPTOD test. Therefore, if the RVPT is analyzed on a microtiter plate reader at a threshold that is darker than the negative control an increased proportion of false positives will occur.

Other researchers have compared the accuracy of detecting pregnancy specific proteins to transrectal ultrasonography. Romano and Larson (2010) compared the accuracy of a PSPB ELISA to transrectal ultrasonography on day 28 post breeding in dairy cattle [21]. They determined sensitivity was 93.9% and specificity was 95.5%. In the current study, all tests (BPT, RVPTOD, RVPTscore, RVPTY/N) had greater sensitivity (>96%), while specificity was lesser in all tests (<90%). They reported positive predictive value, negative predictive value, and accuracy/percent correct were approximately 94.7%. The current study had similar values for the BPT, RVPTscore, and RVPTY/N. However, the RVPTOD had a decreased positive predictive value and accuracy. They also reported a very good kappa score for their test (0.92) that was comparable to the kappa scores from the BPT, RVPTscore, and RVPTY/N in the current study.

Both the BPT and the RVPT were able to determine pregnancy status at 28 days post-AI, or shortly after the conceptus attached to the uterus. Additionally, these tests were able to separate AI pregnancies from natural service pregnancies when AI pregnancies were at or greater than 28 days and natural service pregnancies were less than 25 days. In a production setting several options are available to determine pregnancy status in cattle. However, this study demonstrated that commercially available blood tests can be used to determine AI conception rates as early as 28 days post-AI with a single blood sample without the need for an ultrasound machine or trained technician. Alternatively, a skilled technician can detect pregnancy as early as 30–35 days post breeding by rectal palpation, and in a previous study accuracy at d 28 using a PSPB ELISA was reported to be 94.7% and was similar on days 30 and 35 [21, 22]. However, palpation does not determine viability of the embryo/fetus. Palpation also increases the risk of fetal losses in an early pregnant heifer/cow. Variations in fetal losses among different technicians and palpation methods have also been observed [23]. It has previously been reported that fetal loss was the lowest among all technicians examined when only fluctuation was performed and was greatest when fluctuation and membrane slip was performed. Total percent fetal loss by the three palpation methods was statistically different among the three technicians (4.2%, 6.4%, 9.2%; *P* < 0.025). Thus, a possible alternative to avoid the need for an ultrasound machine and technician, and still decrease the likelihood of embryonic loss at an early stage of gestation is by blood tests.

One of the main factors that influences profitability of beef and dairy herds is embryonic mortality. Among cattle (*Bos taurus*), fertilization rates following artificial insemination range from 89 to 100% [24–28], however, pregnancy rates range from 41 to 75% [29, 30]. Specifically, early embryonic losses, classified as occurring prior to day 24, account for approximately 57% of all pregnancy losses [31], and these animals that lose an embryo/conceptus will either conceive late in the breeding season or fail to conceive during a defined breeding season. Possible reasons for early embryonic loss include: nutritional and environmental factors, chromosomal abnormalities, uterine asynchrony, and inadequate hormone levels [32]. Little is known about specifically when this embryonic loss is occurring, and the mechanism by which it occurs.

Since embryonic mortality can have such an economic impact on cattle operations, the second objective of this study was to determine if blood pregnancy tests could determine beef heifers and cows that are more likely to lose their pregnancy. The large variability in PAG production among individual animals and long half-lives (80–100 d postpartum; [3]) increases the likelihood of false positives. Many studies have investigated circulating pregnancy specific protein profiles following spontaneous or induced embryonic/fetal mortality. Szenci and others [33] examined conceptus protein (bovine pregnancy specific protein B[PSPB], bovine pregnancy associated glycoprotein [PAG1]) profiles in dairy cows that experienced spontaneous embryonic/fetal loss between days 26 and 59 post AI [33]. Following pregnancy loss, PSPB and PAG1 started to decline in most cases. Occurrence of loss could have been predicted successfully in 7 out of 11 cows using established PSPB cut off values, while for PAG1 only 4 out of 11 cows were below the cut off value after conceptus death. This suggests that PAG1 has a longer half-life than PSPB due to differences in sialic acid and carbohydrate content. Pohler and others [34] investigated the accuracy of two PAG assays (2 PAG ELISA, commercial IDEXX test) in predicting embryonic/fetal loss between days 31 and 51 of gestation in dairy cows [34]. They concluded that PAG concentrations that were below 1.8 ng/mL on day 31 resulted in a 95% chance of embryonic/fetal mortality by day 60 of gestation.

Another study examined PAG1 profiles after the induction of late embryonic mortality by prostaglandin and cervical/intraluminal inoculation with *Actinomyces pyogenes* in heifers on days 30–38 of pregnancy [35]. In heifers that were inoculated the half-life of PAG1 was 2.7 to 3.5 days, with plasma PAG1 concentrations started to decrease three days after treatment. While the half-life for heifers treated with prostaglandin was 3.2 to 3.9 days, with plasma PAG1 concentrations starting to decline two to three days following treatment. A study conducted by Giordano and others also focused on changes in PSPB and PAG1 concentrations following prostaglandin and intrauterine infusion of hypertonic solution treatments on day 39.5 post insemination in dairy cows [36]. Serum PSPB and PAG1 concentrations differed among control cows starting on day 1 and day 2.5 of treatment, by day 9.5 post treatment serum levels were similar to non-pregnant cows.

In the current study, beef females that experienced pregnancy loss at some point between the first and second pregnancy diagnosis retrospectively had lower RVPT scores and decreased circulating concentrations of PAGs according to the BPT optical densities. This further supports the idea that the majority of embryonic loss is occurring around the time of attachment (day 20). There may be impaired attachment among beef females that lost a pregnancy, resulting in less PAGs being present in maternal circulation. However, most of these beef females were above the threshold to be considered pregnant on the day of testing. Furthermore, when examining PAG concentrations among heifers and cows, heifers had greater circulating PAG concentrations based on both optical density readings (BPT) and visual scores (RVPT). Previous research has reported similar findings based on age/parity [37,38]. However, cows were scored greater in regard to the RVPT. For the BPT, there was a tendency for an age by pregnancy loss interaction. The detected a greater difference in PAG concentrations among heifers that did and did not lose their pregnancy, compared to cows that did and did not lose their pregnancy. While for the case of the RVPT, both technicians scored heifers that maintained a pregnancy greater than heifers that lost a pregnancy. Unexpectedly, both technicians scored cows that lost their pregnancy greater than cows that maintained their pregnancy. A possible explanation for these results may be in the sensitivity between a plate reader reading optical density and the sensitivity of the human eye to pick up differences in color density.

In conclusion, both pregnancy tests (BPT and RVPT) were accurate at determining pregnancies 28 d post AI, as well as differentiating between natural service and AI pregnancies, and were in extremely high agreement with transrectal ultrasonography. Both pregnancy tests (BPT and RVPT) were also sensitive enough to retrospectively detect differences in circulating PAGs among beef females that experienced pregnancy loss, but prediction with mature cows proves to be difficult. Regardless, most of these beef cows and heifers would have been classified as pregnant at time of sample collection. Therefore, the use of these bovine pregnancy tests to predict pregnancy loss in cattle is not suggested.

## References

[1] Green JA, Xie S, Quan X, Bao B, Gan X, Mathialagan N, et al Pregnancy-associated bovine and ovine glycoproteins exhibit spatially and temporally distinct expression patterns during pregnancy. Biol Reprod. 2000;62(6):1624–31. 10819764 10.1095/biolreprod62.6.1624

[2] Wooding FB. Current topic: the synepitheliochorial placenta of ruminants: binucleate cell fusions and hormone production. Placenta. 1992;13(2):101–13. 1631024 10.1016/0143-4004(92)90025-o

[3] Green JA, Parks TE, Avalle MP, Telugu BP, McLain AL, Peterson AJ, et al The establishment of an ELISA for the detection of pregnancy-associated glycoproteins (PAGs) in the serum of pregnant cows and heifers. Theriogenology. 2005; 63(5):1481–503. 10.1016/j.theriogenology.2004.07.011 15725453

[4] Sasser RG, Ruder CA, Ivani KA, Butler JE, Hamilton WC. Detection of pregnancy by radioimmunoassay of a novel pregnancy-specific protein in serum of cows and a profile of serum concentrations during gestation. Biol Reprod. 1986; 35:936–42. 3814705 10.1095/biolreprod35.4.936

[5] Ruder CA, Sasser RG. Source of bovine pregnancy-specific protein B (bPSPB) during the postpartum period and estimation of half-life of bPSPB. J Anim Sci. 1986; 63(Suppl.), 335 (Abstract).

[6] Kirakofe GH, Wright JM, Schalles RR, Ruder CA, Paris S, Sasser RG. Pregnancy specific protein B in serum of postpartum beef cows. J Anim Sci. 1993; 71: 2199–205. 8376246 10.2527/1993.7182199x

[7] Perry GA, Cushman RA. Invited Review: Use of ultrasonography to make reproductive management The Professional Animal Scientist. 2016;32(2):154–61.

[8] Diskin MG, Sreenan JM. Fertilization and embryonic mortality rates in beef heifers after artificial insemination. J Reprod Fertil. 1980; 59: 463–468. 7431304 10.1530/jrf.0.0590463

[9] Roche JF, Bolandl MP, McGeady TA. Reproductive wastage following artificial insemination of heifers. Vet Rec. 1981; 109: 401–404. 7340073 10.1136/vr.109.18.401

[10] Sreenan JM, Diskin MG. Early embryonic mortality in the cow: its relationship with progesterone concentration. Vet Rec. 1983;112(22):517–21. 6683905 10.1136/vr.112.22.517

[11] Diskin MG, Murphy JJ, Sreenan JM. Embryo survival in dairy cows managed under pastoral conditions. Anim Reprod Sci. 2006; 96: 297–311. 10.1016/j.anireprosci.2006.08.008 16963203

[12] Diskin MG, Parr MH, Morris DG. Embryo death in cattle: an update. Reprod Fertil Dev. 2011;24(1):244–51. 10.1071/RD11914 22394965

[13] Madsen CA, Perry GA, Mogck CL, Daly RF, MacNeil MD, Geary TW. Effects of preovulatory estradiol on embryo survival and pregnancy establishment in beef cows. Anim Reprod Sci. 2015; 158:96–103. 10.1016/j.anireprosci.2015.05.006 26022231

[14] Northrop EJ, Rich JJJ, Cushman RA, McNeel AK, Soares EM, Brooks K, et al Effects of Preovulatory Estradiol on Uterine Environment and Conceptus Survival from Fertilization to Maternal Recognition of Pregnancy. Biol Reprod.2018;99(3): 629–638. 10.1093/biolre/ioy086 29672673

[15] Kasimanickam R, Whittier WD, Tibary A, Inman B. Error in pregnancy diagnosis by pre-rectal palpation in beef cows. Clinical Theriogenology. 2011;3(1):43.

[16] Wimsatt WA. Observations on the morphogenesis, cytochemistry, and significance of the binocleate giant cells of the placenta of ruminants. Am J Anat. 1951; 89(2):233–81. 10.1002/aja.1000890204 14894441

[17] Wathes DC, Wooding FB. An electron microscopic study of implantation in the cow. Am J Anat. 1980;159(3):285–306. 10.1002/aja.1001590305 7211711

[18] Wooding FB. Role of binucleate cells in fetomaternal cell fusion at implantation in the sheep. Am J Anat. 1984;170(2):233–50. 10.1002/aja.1001700208 6465051

[19] Peters AR. Embryo mortality in the cow. Animal Breeding Abstract. 1996;64:587–98.

[20] King GJ, Atkinson BA, Robertson HA. Development of the bovine placentome from days 20 to 29 of gestation.J Reprod Fertil. 1980; 59(1):95–100. 7401049 10.1530/jrf.0.0590095

[21] Romano JE, Larson JE. Accuracy of pregnancy specific protein-B test for early pregnancy diagnosis in dairy cattle. Theriogenology. 2010; 74:932–939. 10.1016/j.theriogenology.2010.04.018 20580072

[22] Ball L. Pregnancy diagnosis in the cow. Current therapy in theriogenology. 1980:229.

[23] Abbitt B, Ball L, Kitto GP, Sitzman CG, Wilgenburg B, Raim LW, et al Effect of three methods of palpation for pregnancy diagnosis per rectum on embryonic and fetal attrition in cows.J Am Vet Med Assoc. 1978;173(8):973–7. 721678

[24] Kidder H, Black W, Wiltbank J, Ulberg L, Casida L. Fertilization Rates and Embryonic Death Rates in Cows Bred to Bulls of Different Levels of Fertility. J Dairy Sci. 1954;37(6):691–7.

[25] Bearden H, Hansel W, Bratton R. Fertilization and embryonic mortality rates of bulls with histories of either low or high fertility in artificial breeding. J Dairy Sci. 1956;39(3):312–8.

[26] Diskin MG, Sreenan JM. Fertilization and embryonic mortality rates in beef heifers after artificial insemination. J Reprod Fertil. 1980;59(2):463–8. 7431304 10.1530/jrf.0.0590463

[27] Maurer RR, Chenault JR. Fertilization failure and embryonic mortality in parous and nonparous beef cattle. J Anim Sci.1983;56(5):1186–9. 6863165 10.2527/jas1983.5651186x

[28] Gayerie de Abreu F, Lamming G, Shaw R, editors. A cytogenetic investigation of early stage bovine embryos: relation with embryo mortality. 10 international congress on animal reproduction and artificial insemination.1984.

[29] Odde KG. A review of synchronization of estrus in postpartum cattle. J Anim Sci. 1990;68(3):817–30. 2180878 10.2527/1990.683817x

[30] Lamb GC, Dahlen CR, Larson JE, Marquezini G, Stevenson JS. Control of the estrous cycle to improve fertility for fixed-time artificial insemination in beef cattle: a review. J ANim Sci. 2010;88(13 Suppl):E181–92. 10.2527/jas.2009-2349 19783709

[31] Inskeep EK, Dailey RA. Embryonic death in cattle. Vet Clin North Am Food Anim Pract. 2005;21(2):437–61. 10.1016/j.cvfa.2005.02.002 15955439

[32] Bridges GA, Mussard ML, Pate JL, Ott TL, Hansen TR, Day ML. Impact of preovulatory estradiol concentrations on conceptus development and uterine gene expression. Anim Reprod Sci. 2012;133(1–2):16–26. 10.1016/j.anireprosci.2012.06.013 22789700

[33] Szenci O, Humblot P, Beckers JF, Sasser G, Sulon J, Baltusen R, et al Plasma profiles of progesterone and conceptus proteins in cows with spontaneous embryonic/fetal mortality as diagnosed by ultrasonography. Vet J. 2000; 159:287–290. 10.1053/tvjl.1999.0399 10775475

[34] Pohler KG, Pereira MH, Lopes FR, Lawrence JC, Keisler DH, Smith MF et al Circulating concentrations of bovine pregnancy-associated glycoproteins and late embryonic mortality in lactating dairy herds. J Dairy Sci. 2016;99(2):1584–94. 10.3168/jds.2015-10192 26709163

[35] Szenci O, Beckers JF, Sulon J, Bevers MM, Borzsonyi L, Fodor L, et al Effect of induction of late embryonic mortality on plasma profiles of pregnancy associated glycoprotein 1 in heifers. Vet J. 2003; 165:307–313. 12672378 10.1016/s1090-0233(02)00180-6

[36] Giordano JO, Guenther JN, Lopes G Jr, Fricke PM. Changes in serum pregnancy-associated glycoprotein, pregnancy-specific protein B, and progesterone concentrations before and after induction of pregnancy loss in lactating dairy cows. J Dairy Sci. 2012;95(2):683–97. 10.3168/jds.2011-4609 22281333

[37] Lobago F, Bekana M, Gustafsson H, Beckers JF, Yohannes G, Aster Y et al Serum profiles of pregnancy-associated glycoprotein, oestrone sulphate and progesterone during gestation and some factors influencing the profiles in Ethiopian Borana and crossbred cattle. Reprod Domest Anim. 2009; 44:685–692. 10.1111/j.1439-0531.2007.01049.x 19055565

[38] Ricci A, Carvalho PD, Amundson MC, Fourdraine RH, Vincenti L, Fricke PM. Factors associated with pregnancy-associated glycoprotein (PAG) levels in plasma and milk of Holstein cows during early pregnancy and their effect on the accuracy of pregnancy diagnosis. J Dairy Sci. 2015;98(4):2502–14. 10.3168/jds.2014-8974 25660740

